# Physical and Oxidative Stability of Emulsions Stabilized with Fractionated Potato Protein Hydrolysates Obtained from Starch Production Side Stream

**DOI:** 10.3390/antiox12081622

**Published:** 2023-08-16

**Authors:** Betül Yesiltas, Pedro J. García-Moreno, Rasmus K. Mikkelsen, Simon Gregersen Echers, Dennis K. Hansen, Mathias Greve-Poulsen, Grethe Hyldig, Egon B. Hansen, Charlotte Jacobsen

**Affiliations:** 1 National Food Institute, Technical University of Denmark, 2800 Kgs. Lyngby, Denmark; rkrmik@food.dtu.dk (R.K.M.); grhy@food.dtu.dk (G.H.); egbh@food.dtu.dk (E.B.H.); 2 Department of Chemical Engineering, University of Granada, 18012 Granada, Spain; pjgarcia@ugr.es; 3 Department of Chemistry and Bioscience, Aalborg University, 2450 Copenhagen, Denmark; sgr@bio.aau.dk; 4 Lihme Protein Solutions, 2750 Ballerup, Denmark; d.hansen@lps-dk.com; 5 KMC Amba, 7330 Brande, Denmark; mgp@kmc.dk

**Keywords:** protein hydrolysate, ultrafiltration, emulsifying peptides, oil-in-water emulsions, antioxidant activity, sensory profile

## Abstract

This work studies the emulsifying and antioxidant properties of potato protein hydrolysates (PPHs) fractions obtained through enzymatic hydrolysis of potato protein using trypsin followed by ultrafiltration. Unfractionated (PPH1) and fractionated (PPH2 as >10 kDa, PPH3 as 10–5 kDa, PPH4 as 5–0.8 kDa, and PPH5 as <0.8 kDa) protein hydrolysates were evaluated. Pendant drop tensiometry and dilatational rheology were applied for determining the ability of PPHs to reduce interfacial tension and affect the viscoelasticity of the interfacial films at the oil–water interface. Peptides >10 kDa showed the highest ability to decrease oil–water interfacial tension. All PPH fractions predominantly provided elastic, weak, and easily stretchable interfaces. PPH2 provided a more rigid interfacial layer than the other hydrolysates. Radical scavenging and metal chelating activities of PPHs were also tested and the highest activities were provided by the unfractionated hydrolysate and the fractions with peptides >5 kDa. Furthermore, the ability of PPHs to form physically and oxidatively stable 5% fish oil-in-water emulsions (pH 7) was investigated during 8-day storage at 20 °C. Our results generally show that the fractions with peptides >5 kDa provided the highest physicochemical stability, followed by the fraction with peptides between 5 and 0.8 kDa. Lastly, promising sensory results with mostly mild attributes were obtained even at protein concentration levels that are higher than needed to obtain functional properties. The more prominent attributes (e.g., bitterness and astringency) were within an acceptable range for PPH3 and PPH4.

## 1. Introduction

Following the growing demand for sustainable food ingredients sourced from natural and sustainable origins, utilization of industrial side streams gained traction as they are often abundant in nutrients and contain bioactive and techno-functional compounds [1]. By harnessing these side streams, the food industry can address the demand while adhering to clean label criteria and considering consumer acceptance [2]. By re-incorporating side streams into food production, the industry can optimize resource utilization, minimize waste, and foster a more sustainable future, where the entire lifecycle of food is maximized. As an emerging application of alternative and natural food ingredients, plant-based protein hydrolysates were investigated for their functional properties in model oil-in-water emulsion systems to gather insight into their properties in real food emulsions [3,4,5,6,7,8]. Oxidatively prone oils, such as fish oil rich in omega-3 polyunsaturated fatty acids, are commonly used in these model systems to increase the rate of oxidation due to the bis-allylic carbons in their molecular structure. Peptides may potentially provide protection against lipid oxidation due to a combination of both surface activity and antioxidant properties. The former is attributed to their amphiphilic character and ability to interact with/adsorb the interface between oil and water in emulsions [9,10]. The antioxidant activity of peptides was suggested to relate to the content of specific amino acids that have the ability to scavenge free radicals (e.g., Cys, Met, and Try) or chelate metals (e.g., His, Glu, and Asp) [11,12].

Despite a modest protein content (1–2%), the magnitude of proteins obtained from the starch industry (~240,000 t/year) makes potatoes a highly relevant source of plant-based protein [13]. The identification and release of abundant proteins/peptides from plant-based sources gained significant attention from the food industry in recent years [9,14]. To meet the increasing demand for sustainable protein-based ingredients from alternative sources, the development of approaches combining advanced mass spectrometry-based proteomics and bioinformatics tools allowed for more time and cost-efficient research [15,16]. Using this approach, we previously identified several potent emulsifier peptides from abundant potato proteins [14,17]. While recent years showed tremendous progress in the fundamental understanding of peptide emulsification, most studies contributing to this were naturally performed on simple model systems and with individually isolated/synthesized peptides [9,10,18,19]. However, the use of purified peptides is not economically viable in the food industry, and thus, it is essential to further investigate and understand how hydrolysates from alternative protein sources may affect the physicochemical stability of oil-in-water emulsions. For instance, trypsin was determined the best candidate for targeted release of validated emulsifier peptides from potato protein and a tryptic hydrolysate was found to show superior emulsifying properties compared to the native protein and a range of hydrolysates obtained through conventional trial-and-error hydrolysis [20]. 

Previous studies reported that the size of the peptides contained in whey protein hydrolysates affects the emulsification ability; therefore, the degree of hydrolysis should be controlled [21,22,23]. In another study, sweet potato protein hydrolysates were obtained by Alcalase under high hydrostatic pressure, which significantly increased the degree of hydrolysis and the <3 kDa fraction content, thereby improving antioxidant activity [7]. Indeed, the size fractionation of the hydrolysates is an important factor to investigate both emulsifying and antioxidant activities in emulsion systems. 

Thus, this study aimed to investigate the emulsifying and antioxidant activities of a potato protein hydrolysate obtained by trypsin and its fractions obtained by ultrafiltration. First, the ability of the unfractionated hydrolysate and fractionated PPHs to reduce interfacial tension, as well as their viscoelasticity of the interfacial layer, was evaluated. Moreover, the radical scavenging and metal chelating activities of the PPHs were measured. In addition, we investigated the ability of PPHs to stabilize low-fat oil-in-water emulsions and control lipid oxidation by following emulsions’ physical and oxidative stability during 8 days of storage. Finally, the sensory properties of the PPHs were evaluated in a solution with a trained panel to map their sensory profile. 

## 2. Materials and Methods

### 2.1. Materials

Potato protein hydrolysate (PPH1) was provided by KMC AmbA (Brande, Denmark). PPH1 was produced by the hydrolysis of denatured potato protein (5 *w*/*v*%) in water using 0.05 *w*/*v*% Trypsin (Pancreatic Trypsin Novo, 6.0 S, 6.0 AU/g) (Novozymes A/S, Bagsværd, Denmark) at pH 8, 50 °C for 24 h. The ratio between protein powder and Trypsin was 100:1 (*w*:*w*). Hydrolysis was carried out in a free-fall pH mode and the change in the pH was tracked every hour. When the pH decreased to 6.6 after 7 h, it was adjusted to 7 and the final pH was noted as 7.45 after 24 h. The enzymatic hydrolysis was followed by enzyme inhibition, centrifugation (2759× *g* for 15 min) (Hermle Z 513 K, Wehingen, Germany), and freezing of the supernatant (PPH1) at −18 °C. The degree of hydrolysis of PPH1 was measured and reported as 3.2%. PPH1 was further fractionated into >10 kDa (PPH2), 5–10 kDa (PPH3), 0.8–5 kDa (PPH4), and <0.8 kDa (PPH5) at Lihme Protein Solutions using sequential ultrafiltration with the following membranes: 10 kDa (K02-E010-05-S, 1.25 m^2^, Repligen, Waltham, MA, USA), 5 kDa (K02-E005-05-S, 1.25 m^2^, Repligen, Waltham, MA, USA), and 0.8 kDa (NFG-2B, 1812F, 0.37 m^2^, Synder, Vacaville, CA, USA). The pump used was KMPi with 8 L/min crossflow and 1 Bar differential pressure (Repligen, Waltham, MA, USA). The protein content of the unfractionated (PPH1) and fractionated (PPH2-PPH5) potato protein hydrolysates was determined by DUMAS method and the results are 2.37, 3.08, 1.28, 1.14, and 0.27 *w*/*v*% for PPH1, PPH2, PPH3, PPH4, and PPH5, respectively. Medium-chain triglycerides (MCT) oil (WITARIX^®^ MCT 60/40) was provided by OI Oleo GmbH (Hamburg, Germany), which was used in interfacial tension and dilatational rheology measurements. Cod liver oil was provided by Vesteraalen’s (Nordland, Norway), which was used in emulsions that were subjected to storage experiments. The peroxide value (PV) of the fish oil was 0.23 ± 0.00 meq. O_2_/kg oil. The fatty acid composition (%, *w*/*w*) of the fish oil was as follows: C14:0 (3.7), C16:0 (9.0), C16:1n-7 (9.6), C18:0 (1.9), C18:1n-9 (15.8), C18:1n-7 (4.5), C18:2n-6 (2.4), C18:3n-3 (1.0), C20:1n-9 (13.7), C20:5n-3 (8.4), C22:1n-11 (5.6), and C22:6n-3 (10.7), which was determined using GC analysis of fatty acid methyl esters (FAME) (AOCS official methods Ce2-66 and 1b-89). The tocopherol content of the fish oil was also analyzed and found as follows: α-tocopherol, 195 ± 3 μg/g oil; β-tocopherol, 5 ± 0 μg/g oil; γ-tocopherol, 116 ± 2 μg/g oil; and δ-tocopherol, 43 ± 1 μg/g oil (AOCS Official Method Ce 8–89). 

### 2.2. Methods

#### 2.2.1. Characterization of the Fractionated PPHs

##### Molecular Weight Distribution of Hydrolysate and Fractions

PPHs were analyzed by size exclusion chromatography (FPLC) using an Äkta Pure chromatography system (GE Healthcare, Brondby, Denmark). A Tricorn 10/300 column was packed with Superdex 30 prep grade (GE Healthcare, Brondby, Denmark). The mobile phase used was 0.050 M potassium phosphate buffer, 0.100 M sodium chloride pH 7.5, at a flow rate of 0.500 mL/min. The absorbance was measured at the 280 nm wavelength.

##### Interfacial Tension and Interfacial Dilatational Rheology

The dilatational surface properties were analyzed using a drop tensiometer (OCA 25, Dataphysics Instruments GmbH, Filderstadt, Germany) at 20 °C. A pendant droplet of the protein fractions with a surface area of 59 mm^2^ was formed at the tip of the needle (diameter 1.83 mm) in MCT oil (Witarix MCT 60/40, IOI Oleo GmbH, Hamburg, Germany) contained in a quartz glass cuvette. The aqueous solutions (pH 7) were prepared at 0.1 g protein/L. The drop contour was monitored and fitted with the Young–Laplace–Gauss equation to obtain the interfacial tension. The change in interfacial tension was monitored for 1 h, followed by a frequency and amplitude sweep. The frequency sweep was performed with frequencies of 0.01, 0.0325, 0.055, 0.0775, and 0.1 Hz at an amplitude of 2.5%. The amplitude sweep was performed with amplitudes of 1, 2, 3, 4, and 5% at a frequency of 0.01 Hz. The droplet was subjected to five oscillatory cycles at every amplitude or frequency. Via oscillatory dilatational deformation experiments (compression and expansion), the complex surface dilatational modulus was calculated from the first harmonic of the Fourier spectrum of the oscillatory interfacial tension signal. The complex surface dilatational modulus is equal to E^*^_d_ = E^′^_d_ + iE^″^_d_, where E^′^_d_ is the dilatational storage modulus (elastic modulus), and E^″^_d_ is the dilatational loss modulus (viscous modulus). The loss tangent is defined as tanΦ = (E^′^_d_/E^″^_d_) [10]. The measurements were conducted in triplicate.

##### In Vitro Antioxidant Activity 

The radical scavenging activity of predicted antioxidant peptides was measured using the DPPH radical scavenging activity method of Yang et al. [24] with some modifications. PPHs were dissolved in water and different concentrations (0.2–50 mM) of the PPH solutions were obtained. Equal parts (100 μL) of 0.1 mM ethanolic DPPH solution and a peptide solution were mixed. The mixture was transferred to the microtiter plate, kept in darkness at room temperature for 30 min, and the absorbance was measured at 515 nm using a spectrophotometer. The scavenging effect was calculated as an inhibition percentage following Equation (1):

DPPH inhibition (%) = (1 − (As − A0)/Ab) × 100,(1)

where As is the sample absorbance (PPHs + DPPH), Ao is the sample blank absorbance (no DPPH), and Ab is the reagent blank absorbance (no PPHs). Results were calculated for 50% inhibition concentration (IC50) and presented in mg/mL. butylated hydroxy toluene (BHT) solution was included as a positive control. All measurements were performed in triplicate.

The iron chelating activity of the PPHs was measured as described in Farvin et al. [25] with some modifications. PPHs were dissolved in water and different concentrations (0.2–50 mM) of PPH solutions were obtained. Concisely, 100 µL of the antioxidant sample was transferred to the microtiter plate and 110 µL of water was added. To start the reaction, 20 µL of 0.5 mM ferrous chloride was added and the plate was shaken. After 3 min, 20 µL of 2.5 mM ferrozine was added and the plate was shaken again. After 10 min in the dark at room temperature, the absorbance was measured at 562 nm. Ethylenediaminetetraacetic acid (EDTA) was employed as a positive control. The chelating capacity and IC50 values were calculated in the same way as for the DPPH inhibition described above.

#### 2.2.2. Production and Physicochemical Stability of Oil-in-Water Emulsions Stabilized with the PPHs

##### Emulsion Production

A total of 250 g of fish oil-in-water emulsions (5 wt%) were stabilized with 0.2 wt.% protein of PPHs. PPH solutions were made in distilled water and kept in a stirrer in a refrigerator in darkness overnight to allow full hydration. The pH of the PPH solutions was checked and adjusted to 7 when necessary, using 1 M NaOH and 1 M HCl. Pre-homogenization was performed using ultra turrax (Ystral, Ballrechten-Dottingen, Germany) for 3 min at 16,000 rpm. Fish oil was added into the aqueous phase within the first minute of mixing. Secondary homogenization was performed using a microfluidizer (M110L Microfluidics, Newton, MA, USA) equipped with a ceramic interaction chamber (CIXC, F20Y, internal dimension 75 μm) at 9 kpsi pressure for three passes. Sodium azide (0.05%) and 50 μM of FeSO_4_ were added to the emulsion obtained after microfluidizer homogenization and it was stirred with a spoon. The final pH of the emulsion was measured. Emulsions were stored for 8 days at 20 °C in darkness. Samples were collected for physical characterization and oxidative stability analyses during storage.

##### Physical Stability of Emulsions

(1) Creaming index

Creaming index was followed throughout the storage on days 0, 1, 2, 4, and 8. The creaming index was calculated based on the equation below:

creaming index (%) = (b/a) × 100,

where a is the total height of the emulsion in the tube, and b is the height of the clear aqueous phase at the bottom of the tube.

(2) Emulsion stability

Emulsions were placed in the Turbiscan cabinet on the same day they were produced and scanned over days (Formulaction, Toulouse, France). The scanning program is shown in Table S1 in the Supplementary Material. Based on the scans, the Turbiscan stability index (TSI) is calculated by the computer. This calculation is based on an integrated algorithm that sums up the evolution of transmittance and backscattering light at every position (h) and based on the scan-to-scan difference over total sample height (h).

$$\mathrm{TSI}\left(t\right)=\frac{1}{N_{h}}\overset{t_{\max}}{\underset{t_{i}=1}{\sum }}\overset{z_{\max}}{\underset{z_{i}=z_{\min}}{\sum }}\left|\mathrm{BST}\left(t_{i},\;z_{i}\right)-\mathrm{BST}\left(t_{i-1},\;z_{i}\right)\right|$$

where N_h_ is the number of height position, t is the time, and z_min_ and z_max_ are the lower and upper height limit. BST is the signal that is taken into account (BS when T < 0.2%, otherwise T). The Turbiscan stability index (TSI) values above 3 are considered unstable while values below 1 are stable according to Formulaction’s webpage. Values in between are in the beginning of destabilization, but mostly non-visual.

(3) Zeta potential

The zeta potential was measured using a Zetasizer Nano ZS (Malvern Instruments, Ltd., Worcestershire, UK) on day 1. Samples were prepared by taking 80 μL emulsion and diluting in 40 mL distilled water and mixing by a vortex. A DTS-1070 disposable folded capillary cell (Malvern Instruments, Ltd., Malvern, UK) was used for loading the samples. Measurements from the same sample were carried out in triplicate using a zeta potential range of (−) 100 to (+) 50 mV, and samples were analyzed with 100 runs at 25 °C.

(4) Droplet size

The droplet size distribution of the emulsions was measured using the Mastersizer 2000 (Malvern Instruments, Ltd., Worcestershire, UK) based on the laser diffraction technique on days 0 and 8 of the storage experiment. Emulsions were diluted in recirculating water set at 3000 rpm until an obscuration of approximately 12–15% was reached. For particle and dispersant, the refractive indices of sunflower oil (1.469) and water (1.330) were used, respectively. Measurements of droplet size were carried out in duplicates or triplicates and they were given as the surface-weighted (D[3,2]) and volume-weighted (D[4,3]) mean diameters.

##### Oxidative Stability of Emulsions

Oxidative stability analyses were carried out on the samples collected on days 0, 1, 2, 5, and 8 during the storage experiment.

(1) Peroxide value

Lipids were extracted using the method described by Bligh and Dyer [26] using a reduced amount of chloroform/methanol (1:1, *w*/*w*). Two extractions were made from each emulsion sample. Peroxide value (PV) was determined on lipid extracts using the colorimetric ferric thiocyanate method at 500 nm according to Shantha and Decker [27] with a spectrophotometer (Shimadzu UV-1280, Holm&Halby, Brøndby, Denmark). Measurements were performed in duplicate.

(2) Tocopherol consumption

Tocopherols were determined using an Agilent 1100 series HPLC (Agilent Technologies, Palo Alto, CA, USA), equipped with a fluorescence detector. Lipid extracts (2 g) from the above-mentioned Bligh and Dyer extraction were weighed and evaporated under nitrogen. Then, lipids were dissolved in 10 mL n-heptane. Heptane solutions (1 mL) were taken into separate vials before injection of an aliquot on a Spherisorb S5 W column (250 × 4.6 mm) (Phase Separation Ltd., Deeside, UK). Elution was performed with an isocratic mixture of n-heptane and 2-propanol (100:0.4, *v*:*v*) at a flow of 1 mL/min injection volume of 20 μL in a column (Waters Spherisorb 3 μm Silica, 4.6 mm I.D. × 150 mm), preceded by a guard column (Waters Spherisorb, 5 μm Silica, 4.6 mm I.D. ×10 mm). A fluorescence detector with excitation wavelength at 290 nm and emission wavelength at 330 nm was used according to the AOCS method [28]. A tocopherol standard mix including α, β, γ, and δ-tocopherol standards was used (Calbiochem 613424). The external quantitative standard was used for calculations. Measurements were performed in duplicate.

(3) Volatile compound formation using dynamic headspace GC-MS

Four grams of emulsion, 5 mL distilled water, and 30 mg of an internal standard (4-methyl-1-pentanol) were placed in a purge bottle. The purge bottle was subjected to a water bath at 45 °C for 30 min under purging with nitrogen (150 mL/min) and the volatile compounds were trapped in Tenax GR tubes. Afterward, the tubes were put into an Automatic Thermal Desorber (ATD-350, Perkin Elmer, Norwalk, CN), which was connected to gas chromatography (GC Agilent 6890 N, Palo Alto, CA, USA; Column: DB-1701, 30 m × 0.25 mm × 1.0 μm). The oven program had an initial temperature of 45 °C for 5 min, increasing with 1.5 °C/min until 55 °C, with 2.5 °C/min until 90 °C, and with 12.0 °C/min until 220 °C, where the temperature was kept for 4 min. A mass spectrometer (MS Agilent 5973, Agilent Technologies, USA; electron ionization mode, 70 eV; mass to charge ratio scan between 30 and 250) was used to separate the individual volatile compounds and these compounds were identified by MS library searches (Wiley 138 K, John Wiley and Sons, Hewlett-Packard, New York, NY, USA). The following standards were selected for quantification: 2-ethyl furan, 1-penten-3-one, pentanal, 1-penten-3-ol, 1-pentanol, hexanal, (E)-2-hexenal, heptanal, (E)-2-heptenal, (Z)-4-heptenal, octanal, (E,E)-2,4-hexadienal, (E,E)-2,4-heptadienal, (E)-2-nonenal, and (E,E)-2,4-decadienal. An ethanolic solution of the standards was prepared and diluted into nine levels (0.1–250 μg/mL) for the calibration curve. Each dilution (30 mg) was added into an emulsion produced in the same way as in one of the emulsions (PPH2), which allows a similar release for standards, as it was for volatile compounds formed in all emulsions. Analysis was run in triplicate.

#### 2.2.3. Sensory Analysis of the PPHs 

The sensory evaluation was performed by a tested and trained objective sensory panel at DTU Food. The panel was specifically trained in objective descriptive analysis and the sensory evaluations were performed in a sensory lab with separated booths under normal daylight and at ambient temperature according to the standards and guidelines for the design and construction of sensory lab to ISO 13300-25492 (2006); ISO 3972 (2011) and ISO 3972 (2011)/Cor 1 (2012); 8586 (2012); and ISO 8589, 2007; NMKL Procedure No. 6, 2023 (Table S3, Supplementary Material).

The first sessions were used to develop a vocabulary to describe the sensory characteristics of the samples. The next three sessions were used to train the sensory panel in measuring and scoring the intensity of the selected sensory attributes. For the training, a set of samples with the different attributes and intensities of the attributes was used to ensure that all experimental conditions were represented. The scale was an unstructured 15 cm line scale with an anchor point at 1.5 cm and 13.5 cm. The samples were poured in plastic cups with lids and stored at 20 °C for one hour before serving. All samples were served in three replicates and in random order with three digital numbers and the assessors were served neutral flat bread and water to clean their mouths between samples. 

The final vocabulary was defined in four categories as listed here: appearance: transparent, color (light/dark); odor: sweet, floral-like, sourish; flavor: sweet, earthy, potato, salty, green, cold tea, umami, bitter; texture: sticky, astringency, and viscous.

#### 2.2.4. Statistical Analysis

Mean value and standard deviations were introduced to Statgraphics 18 (Statistical Graphics Corp., Rockville, MD, USA) for the data analysis. Multiple sample comparison was performed to identify the significant differences between samples at certain sampling days and between sampling days for each sample during storage using Tukey as a post hoc test at *p* < 0.05 significance level. 

## 3. Results and Discussion

### 3.1. Characterization of the Unfractionated Hydrolysate and Their Fractions Obtained by Ultrafiltration

#### 3.1.1. Molecular Weight Distribution 

**Figure 1:**
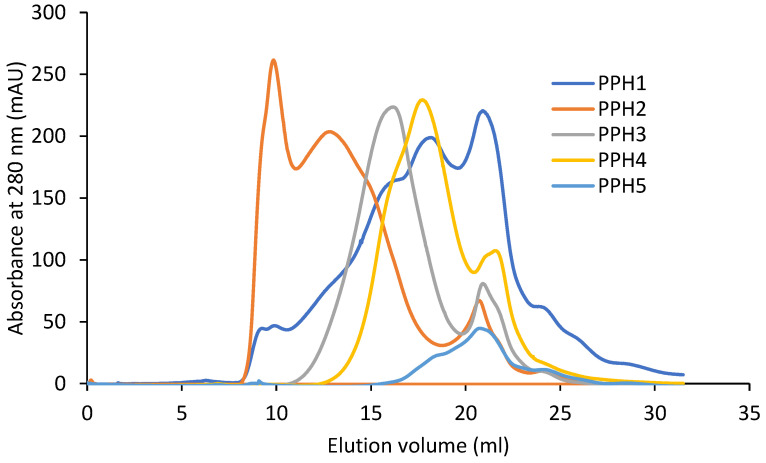
Fast protein liquid chromatography results from potato protein hydrolysates (PPHs). The absorbance was measured at a 280 nm wavelength. PPH1 to PPH5 denotes: unfractionated PPH, >10 kDa, 5–10 kDa, 0.8–5 kDa, and <0.8 kDa, respectively.

The retention volume of the peptides by size exclusion increases from PPH2, PPH3, PPH4, to PPH 5, owing to decreasing peptide sizes (Figure 1). This is in accordance with the decreasing molecular weight cut-off of the membranes that they were retained on during the sequential filtration (10 kDa, 5 kDa, and 0.8 kDa permeate, respectively). PPH1, unfractionated PPH, spans the complete range seen in the fractionated samples. Larger molecules, with a retention volume of less than 11 mL, were exclusively retained in the PPH2 sample.

In contrast, lower-mass peptides (i.e., a retention volume in the range 20–25 mL) seem to be distributed across the different fractions, indicating that lower-mass peptides reside in the retentate post ultracentrifugation. To facilitate more efficient fractionation by size, samples may be passed through the filters with multiple passes by washing in a diafiltration-like manner. However, this would come at the expense of sample dilution and impose substantial additional costs if adapting the process to full-scale production. 

#### 3.1.2. Interfacial Tension and Interfacial Dilatational Rheology

**Figure 2:**
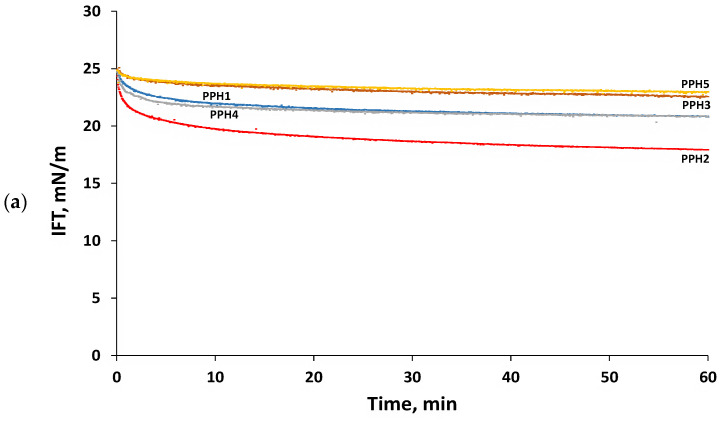

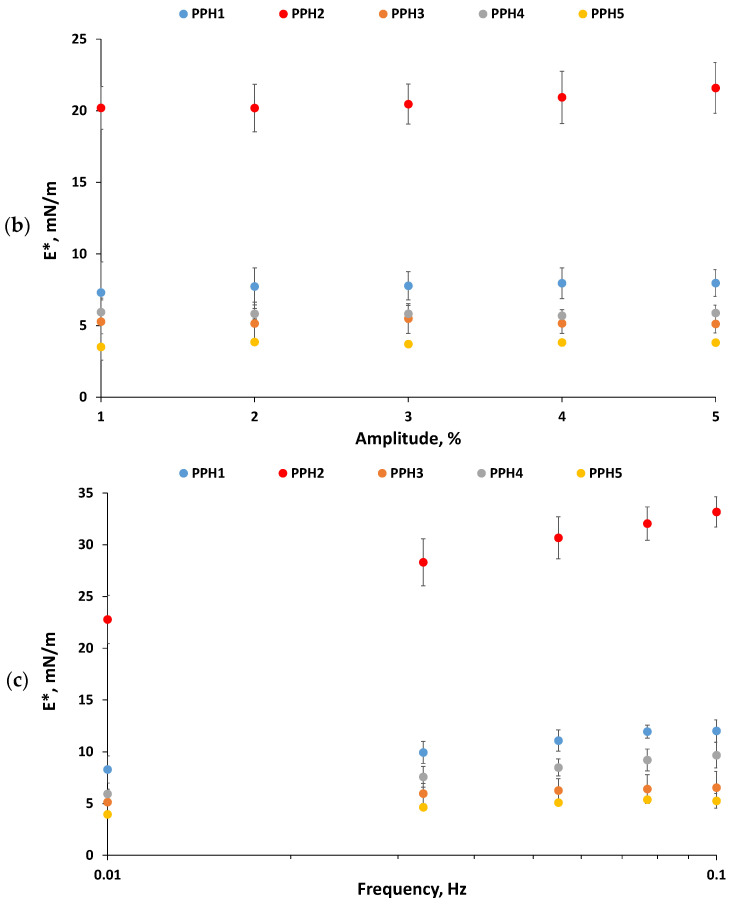
Interfacial properties of PPHs: (**a**) Interfacial tension as a function of time, (**b**) complex surface dilatational modulus (E^*^) as a function of amplitude (1–5%, frequency: 0.01 Hz), and (**c**) complex surface dilatational modulus (E^*^) as a function of frequency (0.005 to 0.1 Hz, amplitude: 2.5%). Measurements were carried out for 0.1 g protein/L PPHs aqueous solutions (pH 7) at 20 °C using MCT oil. The bare MCT oil–water interfacial tension was 26 mN/m. PPH1 to PPH5 denotes: unfractionated PPH, >10 kDa, 5–10 kDa, 0.8–5 kDa, and <0.8 kDa, respectively.

Emulsifying peptides may reduce interfacial tension by (i) diffusing from the bulk aqueous phase to the interface, (ii) adsorbing at the oil/water interface, and (iii) rearranging their structure at the interface to project their hydrophobic residues to the oil phase [16]. Figure 2a shows the reduction in the interfacial tension of the potato protein hydrolysates studied (PPH1) and their fractions obtained by ultrafiltration (PPH2-PPH5). It was observed that the bulk hydrolysate (PPH1) notoriously reduced the interfacial tension during the first 10 min, leveling off at a value of 20.9 ± 0.4 mN/m after 60 min. An identical trend to that observed for the bulk hydrolysate was also found for the hydrolysate fraction PPH4 (5–0.8 kDa). On the other hand, the hydrolysate fractions PPH3 (10–5 kDa) and PPH5 (<0.8 kDa) reduced interfacial tension to a significantly (*p* < 0.05) lower extent when compared to PPH1, reaching a value of 22.9 ± 0.3 mN/m after 60 min. Interestingly, the hydrolysate fraction PPH2 (>10 kDa) led to an initial significantly (*p* < 0.05) faster decrease in interfacial tension when compared to PPH1, leveling off after 25 min and reaching a value of 17.7 ± 0.4 mN/m after 60 min. This finding is in agreement with previous studies reporting that limited protein hydrolysis (degree of hydrolysis, DH, of 3–6%), which results in larger peptides, favored the emulsifying properties of the obtained hydrolysates [20,21,22,23,29]. In fact, PPH2 presents larger peptides than the rest of the fractions with higher potential to present large hydrophobic patches within their structure. These large hydrophobic patches are key to the diffusion and later adsorption of the peptides at the oil/water interface [14,29]. In any case, it should be noted that the interfacial tension values obtained for the potato protein hydrolysate, or its fractions obtained by ultrafiltration were higher than those previously reported for potato emulsifying peptides [10,14]. This finding is ascribed to the complex nature of a hydrolysate and its fractions, where not all peptides present have amphiphilic properties. 

Interfacial dilatational rheology allows the characterization of interfacial layers, providing information on their viscoelasticity, structure, and intermolecular connections [30]. The complex dilatational modulus (E^*^_d_ = E^′^_d_ + iE^″^_d_) determines the resistance of the interfacial layer to external perturbations of its equilibrium state [31]. The elastic part (E^′^_d_) of the complex dilatational modulus indicates the recoverable energy stored in the interface, whereas the viscous part (E^″^_d_) represents the energy lost through relaxation processes [32]. The hydrolysate and all the ultrafiltration fractions formed predominantly elastic interfacial layers at the tested frequencies and amplitudes, having values of E^′^_d_ markedly larger than E^″^_d_ as well as low values of phase angles (Φ ≤ 26°). This finding denotes that all the peptide-based interfacial layers evaluated showed a gel-like structure with peptide–peptide interactions. The complex dilatational modulus observed for the interfaces studied ranged from 3.5 ± 0.9 to 33.2 ± 1.5 mN/m (Figure 2b,c), which is in line with previously reported values for whey and fish protein hydrolysates [21,31]. All the interfacial layers were amplitude-independent in the range 1–5%, denoting that they were within the linear viscoelastic regime (LVE). Moreover, the lack of change in the interfacial moduli with increasing amplitude (Figure 2b) suggests weak and easily stretchable interfaces that did not suffer a disruption in their microstructure in this amplitude range [10]. Figure 2c shows that the elasticity of all the interfaces, but particularly PPH2, PPH1, and PPH4, increased when increasing frequency. This is due to the shorter time needed to adapt to the deformation via the diffusional exchange of peptides with the aqueous phase or relaxation processes within the network [31]. Interestingly, Figure 2b,c shows that PPH2 formed the most rigid interfacial layer (e.g., highest E^*^_d_), followed by the unfractionated hydrolysate (PPH1) and PPH3-PPH4, with PPH5 presenting the lowest E^*^_d_ (Figure 2b,c). Indeed, PPH2 (>10 kDa) presented large peptides and unhydrolyzed proteins that re-arrange at the interface, facilitating intermolecular connections that result in an interface with higher elasticity and strength [21]. On the contrary, low molecular weight peptides present in PPH5 (<0.8 kDa) were not able to interconnect at the interface, leading to the weakest interface.

#### 3.1.3. In Vitro Antioxidant Activity

**Figure 3:**
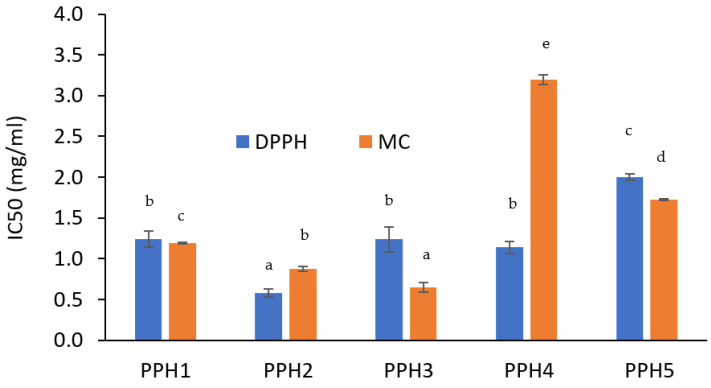
Half maximal inhibitory concentration (IC50) of PPHs is shown for DPPH radical scavenging (blue) and ferrozine metal chelating (MC) (orange) activities. Letters a–e indicate significant difference between samples (*p* < 0.05). PPH1 to PPH5 denotes: unfractionated PPH, >10 kDa, 5–10 kDa, 0.8–5 kDa, and <0.8 kDa, respectively.

Based on the DPPH radical scavenging activity results, PPH2 exhibited a significantly lower IC50 value compared to PPH1, PPH3, and PPH4 (Figure 3). PPH5 was found to be the least effective to scavenge radicals. On the other hand, based on the ferrozine assay, PPH3 was better at chelating metals followed by PPH2, PPH1, PPH5, and PPH4 (Figure 3). This is a different trend compared to the radical scavenging activity of the same PPHs, which could be attributed to the impact of peptide size on their ability to act as antioxidants via different mechanisms. In a previous study, peptide size was reported to affect the potency of peptides to retard lipid oxidation, where shorter peptides are generally attributed to higher scavenging and lipid peroxidation inhibition activities compared to long-chain peptides [16]. Ren et al. outlined that peptide chains containing <3 kDa peptides were more effective scavengers of hydroxyl radicals and inhibitors of lipid peroxidation when compared to chains containing >3 kDa peptides [33]. Therefore, it can be inferred that shorter peptide chains have higher DPPH radical scavenging activity than longer peptide chains. However, our results show that the smallest fraction (as for PPH5) had the highest IC50 value, indicating the lowest radical scavenging activity, which suggests that the smaller peptides (e.g., <0.8 kDa) should be reconsidered and tested for their inferior activity compared to the fractions with a larger size, but still considered short. Indeed, PPH4, containing peptides between 0.8 and 5 kDa, needed to be used at a lower concentration than PPH5 to provide 50% DPPH inhibition, which presents them as a more promising fraction, followed by PPH2 (>10 kDa). On the other hand, it should be borne in mind that the inorganic salt content present in the different fractions could impact the DPPH color-quenching activity and interfere with the assay [34]. 

Metal chelation activity of PPH was also attributed to smaller peptides in a previous study, generally having a size of less than 1 kDa [35]. However, this was not the case for the PPH5 (<0.8 kDa) in our study. PPHs higher than 5 kDa had lower IC50 concentrations, indicating their better metal chelation activity. Indeed, the size of the peptides cannot be considered as the only factor to control the antioxidant activity. There are other factors, such as peptide charge-to-mass ratio, conformation, amino acid composition, as well as the presence of AA residues with carboxyl or imidazole groups as addressed by García-Moreno et al. [14]. Nevertheless, the antioxidant activity of emulsifying hydrolysates should be further tested in model emulsion systems where oxidation is evaluated by advanced techniques such as GC-MS. 

### 3.2. Physicochemical Stability of Fish Oil-in-Water Emulsions Stabilized with PPHs 

#### 3.2.1. Physical Stability of Emulsions

##### Emulsion Stability 

**Figure 4:**
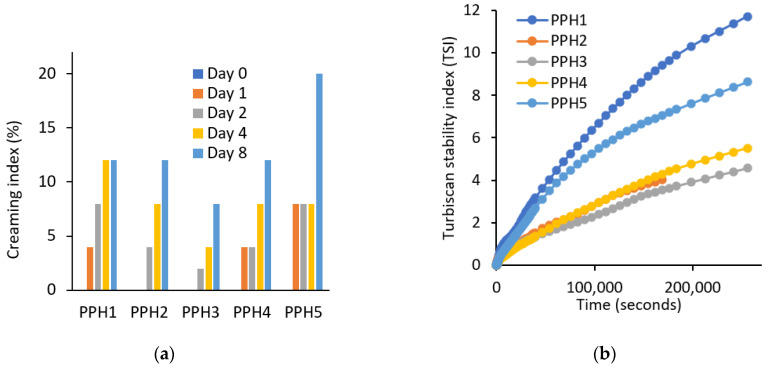
Physical stability of emulsions produced with PPHs; (**a**) creaming (%) and (**b**) Turbiscan stability index. PPH1 to PPH5 denotes: unfractionated PPH, >10 kDa, 5–10 kDa, 0.8–5 kDa, and <0.8 kDa, respectively.

According to the creaming results, samples are ranked from the most physically stable to the least in the following order: PPH3 > PPH2 > PPH4 > PPH1 > PPH5 (Figure 4a). PPH5 emulsion was the least stable, which is expected, as this fraction contains the smallest peptides that are not large enough to adsorb at the oil–water interface, as also shown by the interfacial tension measurements (Figure 2a). Low molecular peptides tend to have weak interaction with each other and cannot form a strong interfacial layer as stated in Section 3.1.3. Similar to the creaming results, the Turbiscan stability index (TSI) values indicate that the emulsions from the most stable to the least stable can be listed as the following: PPH3, PPH2, PPH4, PPH5, and PPH1 (Figure 4b). The only difference was that the PPH1, unfractionated hydrolysate, was the least stable in terms of not only creaming, but also other instability phenomena, such as sedimentation, flocculation, and phase separation. This could be attributed to the inferior surface activity of peptides <0.8 kDa contained in PPH1. 

Overall, based on the two stability indicators, the creaming index and TSI discussed, emulsions stabilized with PPH2, PPH3, and PPH4 (fractions between 10 and 0.8 kDa) were more physically stable compared to PPH1 (unfractionated) and PPH5 (<0.8 kDa). As concluded in Section 3.1.2, PPH2 led to the highest reduction in interfacial tension and to the interfacial film with the highest viscoelasticity, denoting the ability to form small oil droplets and a strong interface that provides resistance against physical destabilization phenomena.

##### Zeta Potential and Droplet Size

Zeta potential and droplet size results are shown in Table 1. The zeta potential value of the PPH5 emulsion was significantly lower compared to the other PPH fractions, indicating that the repulsion between droplets could be lower; however, the net value is still higher than 30 mV, which is considered to provide sufficient repulsion to prevent droplet merging [36]. The rest of the PPHs (PPH1 to PPH4) had a net zeta potential of around −60 mV, which is also in line with the findings in a previous study, in which 5% oil-in-water emulsion was stabilized with a synthetic peptide (namely, g1) derived from potato protein, reporting a value of −62 mV at pH 7 [16]. In another study, zeta potential values were reported as 36.3 ± 0.06, −46.8 ± 0.72 and −44.0 ± 0.60 mV for the 5% fish oil-in-water emulsions stabilized with blue witting hydrolysate (pH 2), soy hydrolysate (pH 8), and whey protein hydrolysate (pH 8), respectively [37]. The absolute zeta potential values in the current study were higher (Table 1) compared to the emulsions containing the different hydrolysates from fish, soy, and milk sources. Another study reported on the concentration effect of the protein emulsifier where the maximum zeta potential was obtained for whey protein-stabilized emulsions (pH 7.8) at the concentrations of 0.5 wt% and 1 wt% with −25 mV and slightly declined to −21 and −19 mV at the concentrations of 2 and 4 wt%, respectively [21]. The concentration of the protein hydrolysate also has an impact on the magnitude of the zeta potential values. 

Emulsions stabilized with PPH3 provided the formation of the smallest droplets compared to the rest of the PPHs based on D[3,2], and this was followed by PPH2 and PPH4 (Table 1). Based on the D[4,3] results, the smallest droplets were obtained for PPH3 and PPH2, followed by PPH4 (Table 1). Overall, emulsions stabilized with PPH fractions with peptides larger than 5 kDa (PPH3 and PPH2) provided smaller droplets (Table 1) and better physical stability (Figure 4) on day 1 of the storage experiment compared to the emulsions stabilized with unfractionated PPH (PPH1) and peptides smaller than 5 kDa. This could be due to better coverage of the oil–water interface with the larger peptides compared to the smaller ones. Indeed, in a previous study, it was reported for whey protein hydrolysate-stabilized emulsions that the peptides larger than 5 kDa were more predominant at the oil–water interface compared to smaller peptides (<5 kDa) [21]. Similarly, results obtained from emulsions stabilized with fava bean hydrolysates at two different levels of degrees of hydrolysis (8.4 and 15.6%) showed that emulsions with the lower degree of hydrolysis yielding larger peptides provided more favorable emulsifying activity [8]. 

In the previously mentioned study on the synthetic potato-derived peptide, g1, authors reported similar droplet sizes obtained for both the D[3,2] and D[4,3] as 151 nm and 250 nm, respectively [16]. However, the synthetic peptide, g1, was much more stable throughout the storage, with no significant increase in both droplet size values. This could be due to there being less complexity when having one compound for the synthetic peptide compared to a hydrolysate, where a plethora of peptides are present and compete for adsorption at the oil–water interface and form a stable layer. This could be attributed to the differences in the adsorption behavior and self-assembling (e.g., based on their secondary structure and amphipathic properties) of the different peptides in the hydrolysates at the oil–water interface [14,38].

#### 3.2.2. Oxidative Stability of Emulsions

**Figure 5:**
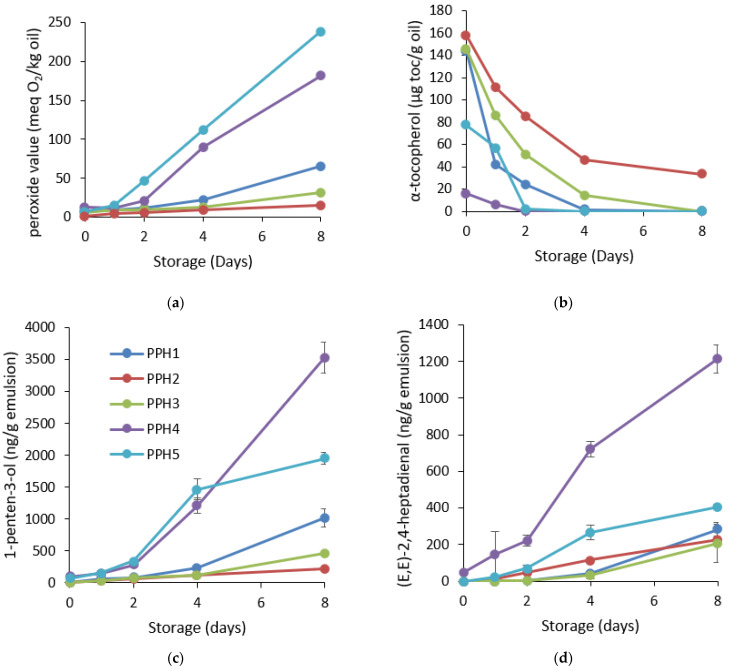
Oxidative stability of the emulsions containing PPHs: (**a**) formation of hydroperoxides; (**b**) consumption of α-tocopherols; (**c**) development of 1-penten-3-ol; and (**d**) development of (*E,E*)-2,4-heptadienal during 8 days of storage at room temperature in darkness. The PV values lower than 50 have less than a 10% average coefficient of variation, and for the values above 50, the average coefficient of variation is less than 5%. For tocopherols, the average coefficient of variation is less than 5%. PPH1 to PPH5 denotes: unfractionated PPH, >10 kDa, 5–10 kDa, 0.8–5 kDa, and <0.8 kDa, respectively.

Emulsions containing PPH2 provided the highest protection against the formation of hydroperoxides throughout the 8 days of storage as indicated by the peroxide value (Figure 5a). The lag phase was the shortest for the emulsions containing PPH1 or PPH4. PV for these emulsions already started to increase significantly after day 4. Overall, if we look at the status of oxidation on the last day of the storage (day 8), PPH2 had the lowest concentration of hydroperoxides, followed by PPH3 and PPH1, whereas emulsions containing PPH4 and PPH5 were the least stable ones (Figure 5a). Similarly, the consumption of α-tocopherol, which is a natural antioxidant in fish oil, in these emulsions followed the opposite order, where emulsions PPH4 and PPH5 depleted significantly earlier than emulsions containing PPH1, PPH2, and PPH3 (Figure 5b). Indeed, the consumption of α-tocopherol followed the same trend as the formation of peroxide values. Moreover, emulsions with PPH4 or PPH5 had significantly lower levels of α-tocopherols already at day 0, indicating that the oxidation was happening already during the emulsification process.

Among the formed secondary oxidation volatile compounds, 1-penten-3-ol and (*E,E*)-2,4-heptadienal were selected as omega-3-derived polyunsaturated fatty acids developed to the highest concentrations during the 8 days of storage. The trend between these 2 volatile compounds was similar with some slight differences in the lag phases of the different emulsions. Formation of 1-penten-3-ol indicated that emulsions containing PPH1, PPH2, and PPH4 had a lag phase of 4 days, whereas PPH3 and PPH5 had a lag phase of 2 days (Figure 5c, Table S2). On the last day of storage, the concentration of 1-penten-3-ol was significantly lower (*p* < 0.05) for PPH2 and PPH3, followed by PPH1, PPH5 and PPH4. Similarly, the formation of (*E-E*)-2,4-heptadienal was significantly slower (*p* < 0.05) for the emulsions produced with PPH1 and PPH3 starting after day 4 (Figure 5d, Table S2). The emulsion containing PPH4 had significantly (*p* < 0.05) higher amounts of (*E-E*)-2,4-heptadienal after day 2, followed by PPH2 and PPH5 starting after day 1. For this specific volatile compound, the emulsion containing PPH4 was the most oxidized at the end of the storage by around 6 folds in comparison to emulsions containing PPH 3 and PPH2. In a previous study, where the synthetic emulsifier peptides were used for stabilizing 5% fish oil-in-water emulsions, the volatile compounds *(E,E)*-2,4-heptadienal, *(E,E)*-2,4-decadienal, 2-ethyl furan, and 1-penten-3-ol had the highest concentration during 10-day storage, indicating a similar profile of secondary lipid oxidation markers in fish oil emulsions [16]. The concentrations of the volatile compounds developed for emulsions containing synthetic peptides were comparable to the current study, where the emulsions contained PPHs. When all the detected volatiles were screened, the emulsions containing PPH2, PPH3, and PPH1 were the most oxidatively stable, followed by the emulsions containing PPH5 and PPH4 (Figure 5 and Figure S1). Fish oil-in-water emulsions stabilized with sardine or small-spotted catshark hydrolysates also reported high concentrations of 1-penten-3-ol and (E,E)-2,4-heptadienal at day 7, and the levels were comparable with PPH2 and PPH3 in the current study [23].

Overall, based on the formation of hydroperoxides and secondary volatile oxidation products and consumption of tocopherols, PPH2 (>10 kDa) had the lowest oxidation levels, followed by PPH3 (10–5 kDa), indicating that the PPH fractions higher than 5 kDa were the most effective for protection against lipid oxidation and performed better in terms of providing oxidative stability, whereas PPH4, as the fraction 5–0.8 kDa, provided the lowest oxidative stability among others, followed by PPH5, the fraction lower than 0.8 kDa. These results are also in line with the in vitro antioxidant activity of the same PPHs. The DPPH radical scavenging activity of the PPH2 (>10 kDa) had higher activity than the PPH3 and PPH4, which contained peptides between 0.8 and 10 kDa, and the lowest activity was obtained for the peptides <0.8 kDa (PPH5). Similarly, the metal chelating activity of the peptides between 5 and 10 kDa (PPH3) was significantly higher followed by the peptides >10 kDa (PPH2), <0.8 kDa (PPH5), and 0.8–5 kDa (PPH4), respectively. The better oxidative stability of the emulsions containing larger peptides of potatoes can potentially be attributed to the antioxidant activity of unadsorbed peptides in the bulk aqueous phase of the emulsions. Specifically, the negatively charged metal chelator peptides at pH 7 may inhibit oxidation reactions by attracting and chelating positively charged metal ions in the aqueous phase. Moreover, peptides efficiently scavenge radicals at the oil–water interface where lipid oxidation is initiated due to having prooxidants in close proximity to lipids [23,39].

The physical stability of emulsions is greatly influenced by the interfacial tension and interfacial dilatational rheology, which are critical factors impacting lipid oxidation [40]. According to Section 3.1.2 and Section 3.2.1 of the study, when emulsifying peptides in the PPHs are introduced into the emulsion, they reduce interfacial tension by adsorbing at the interface between oil and water and reorganizing their structure. Peptides with a molecular weight larger than 5 kDa, such as PPH2 and PPH3, exhibit superior interfacial properties. They effectively decrease interfacial tension and/or display higher viscoelasticity. Consequently, these peptides promote the formation of stable emulsions with smaller droplet sizes, indicating the presence of a strong interface that resists physical destabilization. The physical stability of the emulsion is closely linked to its oxidative stability. Emulsions stabilized with PPH2 and PPH3 demonstrate higher physical stability and smaller droplet sizes, which can contribute to improved oxidative stability. The larger peptides offer better coverage of the oil–water interface, potentially reducing lipid oxidation reactions at the interface by providing both chemical protection through radical scavenging or metal chelating activities and a physical barrier by forming a tightly packed interface. This is likely due to the more complex arrangements of the hydrolysates at the oil–water interface, which prevent interaction with iron. However, this may differ for emulsions stabilized with pure synthetic peptides, as previously reported [10]. Additionally, iron diffusion through the interface is minimal since its solubility in water is higher [41]. Thus, the key is to prevent the interaction between iron in the aqueous phase and polar lipid hydroperoxides at the oil–water interface. Consequently, emulsions stabilized with PPHs larger than 5 kDa, particularly PPH2 and PPH3, exhibit enhanced physical stability, smaller droplet sizes, and potentially improved oxidative stability compared to those stabilized with smaller peptides (PPH4 and PPH5) or unfractionated PPH (PPH1).

In summary, emulsions stabilized with PPHs larger than 5 kDa demonstrated enhanced physical and oxidative stability, together with their antioxidant activity, PPH fractions are promising candidates for applications requiring stable emulsions with enhanced resistance to lipid oxidation.

### 3.3. Sensory Analysis on the PPHs

**Figure 6:**
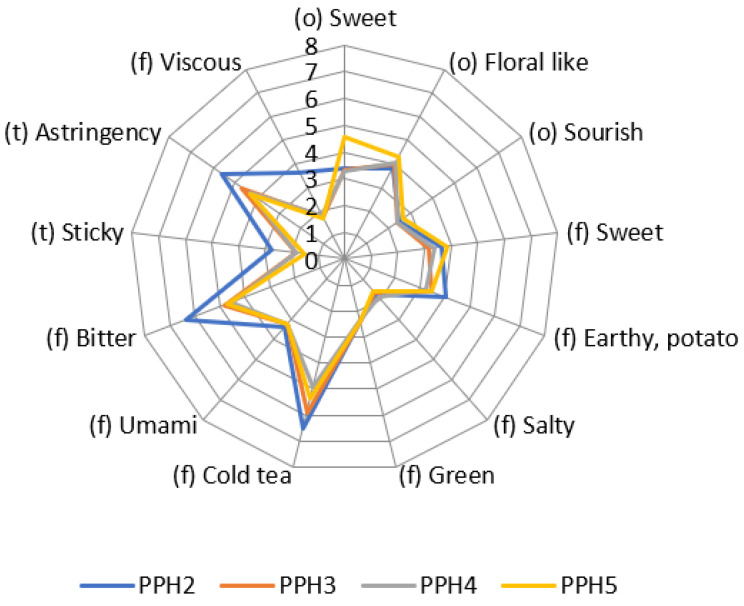
Sensory analysis of the size fractionated PPH solutions. The numbers from 0 to 8 indicate the increasing intensity of the different attributes. PPH2 to PPH5 denotes: >10 kDa, 5–10 kDa, 0.8–5 kDa, and <0.8 kDa, respectively.

Size-fractionated potato protein hydrolysates were measured with objective sensory analysis as hydrolysate solutions with a tested and trained panel. Results are shown in a spider plot in Figure 6. For PPH3 and PPH4, the more prominent attributes were astringency, bitter and cold tea, which were not in a very high intensity and may be in an acceptable range when used in a food product. It should be noted that PPH2 had a higher bitter flavor and astringent taste compared to PPH3, PPH4, and PPH5.

Peptides obtained through protein hydrolysis often possess a bitter taste, which hampers their usefulness in food products [42]. To address this, researchers investigated the impact of different proteolytic treatments on the bitterness of pea protein hydrolysates. The study revealed that among the tested hydrolysates, Alcalase hydrolysate exhibited the highest level of bitterness, while papain and α-chymotrypsin hydrolysates demonstrated the lowest bitterness [43]. Consequently, it is crucial to assess the bitterness intensity and select appropriate fractions of protein hydrolysates for incorporation into food products. Notably, PPH3 and PPH4 are better suited than PPH2 for foods where the final product’s taste could potentially be affected by bitterness. However, it is important to conduct consumer studies to evaluate the acceptance of food products containing protein hydrolysates, as this largely depends on the specific food matrix used. Moreover, masking flavors may be necessary for many plant proteins and protein hydrolysates to achieve desirable sensory attributes [44,45,46,47]. Thus, additional research is needed to determine effective strategies for mitigating the bitter taste and ensuring the acceptability of food products incorporating protein hydrolysates.

## 4. Conclusions

Emulsions produced with unfractionated and size-fractionated potato protein hydrolysates (PPHs) obtained by targeted hydrolysis with trypsin provided physiochemically stable emulsions. Different fractions showed differences in their in vitro antioxidant activity in terms of radical scavenging and metal chelating activities, where PPH2 (>10 kDa) had the best radical scavenging activity and PPH3 (5–10 kDa) had the best metal chelating activity. PPH2 (>10 kDa) had significantly stronger peptide–peptide interactions leading to the formation of more rigid and stable oil–water interfacial layers. Overall, emulsions containing PPHs higher than 5 kDa resulted in higher physical and oxidative stability during 8-day storage, indicating that the size fractionation influences techno-functional properties. This is crucial in the utilization of these ingredients as the fractions can be selected based on their best performance in the required functional property as antioxidants, emulsifiers, or both. Promising sensory results were also obtained at a higher protein concentration than needed to obtain expected functional properties.

## Figures and Tables

**Table 1:** Surface-weighted mean diameter (D[3,2]), volume-weighted mean diameter (D[4,3]), and zeta potential of potato protein hydrolysates. PPH1 to PPH5 denotes: unfractionated PPH, >10 kDa, 5–10 kDa, 0.8–5 kDa, and <0.8 kDa, respectively.

Sample Code	ξ-Potential (mV) Day 1	D[3,2] (µm) Day 1	D[3,2] (µm) Day 8	D[4,3] (µm) Day 1	D[4,3] (µm) Day 8
PPH1	−59.6 ± 1.3 ^a^	0.217 ± 0.002 ^d^	0.327 ± 0.000 ^d^	3.30 ± 0.04 ^c^	4.77 ± 0.15 ^c^
PPH2	−61.0 ± 2.0 ^a^	0.143 ± 0.000 ^b^	0.188 ± 0.008 ^b^	0.33 ± 0.01 ^a^	2.71 ± 1.85 ^b^
PPH3	−60.2 ± 1.4 ^a^	0.134 ± 0.001 ^a^	0.159 ± 0.001 ^a^	0.26 ± 0.00 ^a^	0.65 ± 0.00 ^a^
PPH4	−60.7 ± 2.2 ^a^	0.193 ± 0.003 ^c^	0.243 ± 0.000 ^c^	1.73 ± 0.21 ^b^	2.02 ± 0.20 ^ab^
PPH5	−53.2 ± 0.3 ^b^	0.334 ± 0.006 ^e^	0.367 ± 0.002 ^e^	4.49 ± 0.88 ^d^	6.73 ± 0.28 ^d^

^a–e^ indicates significant differences between samples at a certain sampling day (*p* < 0.05).

## Data Availability

Data is available on request.

## Supplementary Materials

The following supporting information can be downloaded at: https://www.mdpi.com/article/10.3390/antiox12081622/s1, Table S1: Scanning program for the Turbiscan; Table S2: Statistical analysis for the (a) formation of hydroperoxides; (b) consumption of α-tocopherols; (c) development of 1-penten-3-ol and (d) development of (*E*,*E*)-2,4-heptadienal; Figure S1: The development of volatile compounds during 8 days of storage; Table S3: References for sensory analysis.
