# Supplementary material for: Physical and Oxidative Stability of Emulsions Stabilized with Fractionated Potato Protein Hydrolysates Obtained from Starch Production Side Stream
Source: Antioxidants (Basel). 2023 Aug 16;12(8):1622. doi: 10.3390/antiox12081622 (PMC10451251; doi:10.3390/antiox12081622)
Supplement: Supplementary file 1 [file antioxidants-12-01622-s001.zip › antioxidants-2508079-supplementary.pdf]

Supplementary materials for the article

Physical and Oxidative Stability of Emulsions Stabilized with Fractionated Potato Protein Hydrolysates Obtained from Starch Production Side Stream

Betül Yesiltas [1,*], Pedro J. García-Moreno [2], Rasmus K. Mikkelsen [1], Simon Gregersen Echers [3], Dennis K. Hansen [4], Mathias Greve-Poulsen [5], Grethe Hyldig [1], Egon B. Hansen [1] and Charlotte Jacobsen [1,*]

Table S1. Scanning program for the Turbiscan

#scans	Interval	time
31	1 min	40 min
31	10 min	5h20min
16	20 min	7h
10	2h	20h
10	2h	22h
7	4h	1d2min
	Total	
105 scans		3d7h2min

Table S2. Statistical analysis for the (a) formation of hydroperoxides; (b) consumption of α-tocopherols; (c) development of 1-penten-3-ol and (d) development of *(E,E)*-2,4-heptadienal. Letters significant differences between samples at a certain sampling day ($p < 0.05$).

**(a) PV**	**Day 0**	**Day 1**	**Day 2**	**Day 4**	**Day 8**
PPH1	7.5±1.1 [a]	9.0±0.3 [a]	12.0±0.2 [a]	22.1±0.0 [b]	65.1±8.3 [c,C]
PPH2	1.2±0.1 [a]	5.0±0.0 [b]	5.6±0.1 [b]	8.9±0.7 [c]	15.1±0.0 [d,A]
PPH3	5.7±0.3 [a]	9.2±0.5 [b]	9.4±0.1 [b]	13.0±0.4 [c]	31.7±1.9 [d,B]
PPH4	12.8±3.4 [a]	11.8±0.4 [a]	21.2±0.8 [a]	90.3±2.2 [b]	181.1±9.5 [c,D]
PPH5	6.9±0.7 [a]	14.9±1.1 [a]	46.5±0.5 [b]	112.5±6.9 [c]	238.1± - [d,E]
**(b) α-tocopherol**	**Day 0**	**Day 1**	**Day 2**	**Day 4**	**Day 8**
PPH1	144.1±1.7 [d]	42.1±0.4 [c]	24.5±1.4 [b]	1.7±0.9 [a]	0.0±0.0 [a,C]
PPH2	157.9±0.9 [e]	111.1±1.5 [d]	85.0±0.0 [c]	46.2±1.5 [b]	33.1±9.3 [a,C]
PPH3	146.1±2.5 [e]	86.1±0.1 [d]	51.1±0.1 [c]	14.6±3.6 [b]	0.0±0.0 [a,C]
PPH4	16.0±7.1 [b]	6.2±3.0 [a]	0.0±0.0 [a]	0.0±0.0 [a]	0.0±0.0 [a,A]
PPH5	77.5±9.6 [c]	57.3±7.5 [b]	1.7±0.4 [a]	0.0±0.0 [a]	0.0± - [a,B]
**(c) 1-Penten-3-ol**	**Day 0**	**Day 1**	**Day 2**	**Day 4**	**Day 8**
PPH1	3.0±2.5 [a]	67.1±13.9 [a]	78.9±23.5 [a]	228.6±7.4 [b]	1016.6±142.0 [c,B]
PPH2	0.0±0.0 [a]	32.1±3.4 [a]	62.6±4.3 [ab]	125.2±15.1 [b]	217.4±55.9 [c,A]
PPH3	0.0±0.0 [a]	38.4±6.8 [ab]	73.4±16.7 [b]	124.7±26.9 [c]	462.5±8.3 [d,A]

PPH4	97.5±8.3 a	148.1±25.7 a	281.0±10.9 a	1206.7±122.1 b	3523.5±242.9 c,D
PPH5	65.9±9.9 a	156.0±38.4 ab	343.2±31.1 b	1465.9±164.6 c	1950.6±92.8 d,C
**(d) (E,E)-2,4-heptadienal**	**Day 0**	**Day 1**	**Day 2**	**Day 4**	**Day 8**
PPH1	0.0±0.0 a	0.0±0.0 a	2.3±3.9 a	41.9±6.3 a	283.6±35.5 b,AB
PPH2	0.0±0.0 a	13.2±2.6 ab	49.0±30.3 b	115.4±15.3 c	225.0±5.3 d,A
PPH3	0.0±0.0 a	0.3±0.5 a	2.0±2.1 a	32.4±19.2 a	206.9±105.0 b,A
PPH4	49.1±12.1 a	145.2±126.4 a	221.6±31.9 a	723.0±42.3 b	1214.1±77.7 c,C
PPH5	0.0±0.0 a	22.9±5.5 ab	69.8±19.2 b	264.2±40.1 c	407.3±13.3 d,B

Figure S1. The development of volatile compounds during 8 days of storage

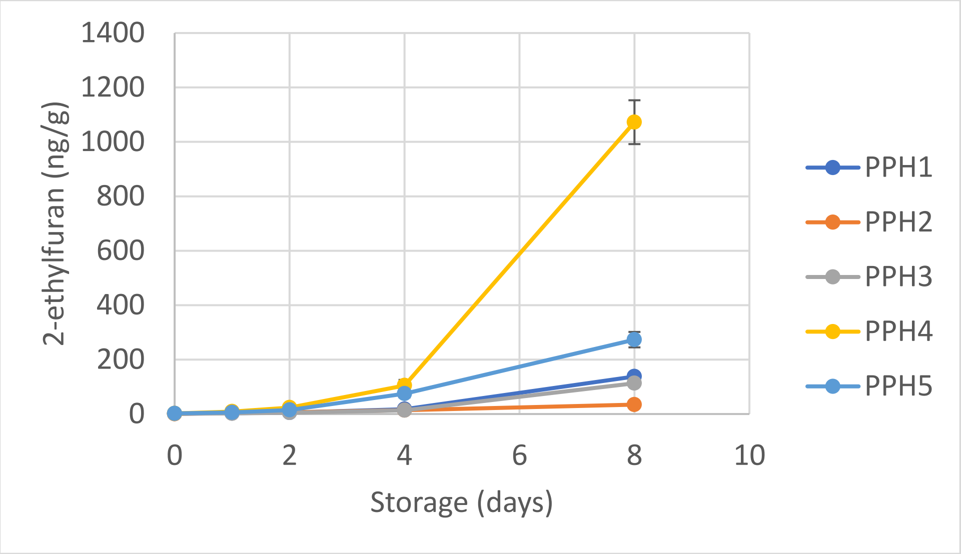

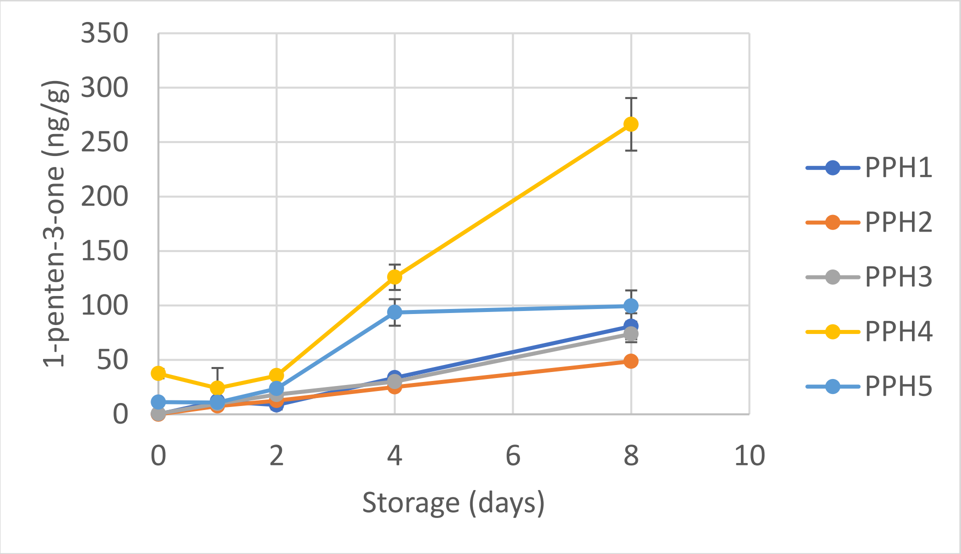

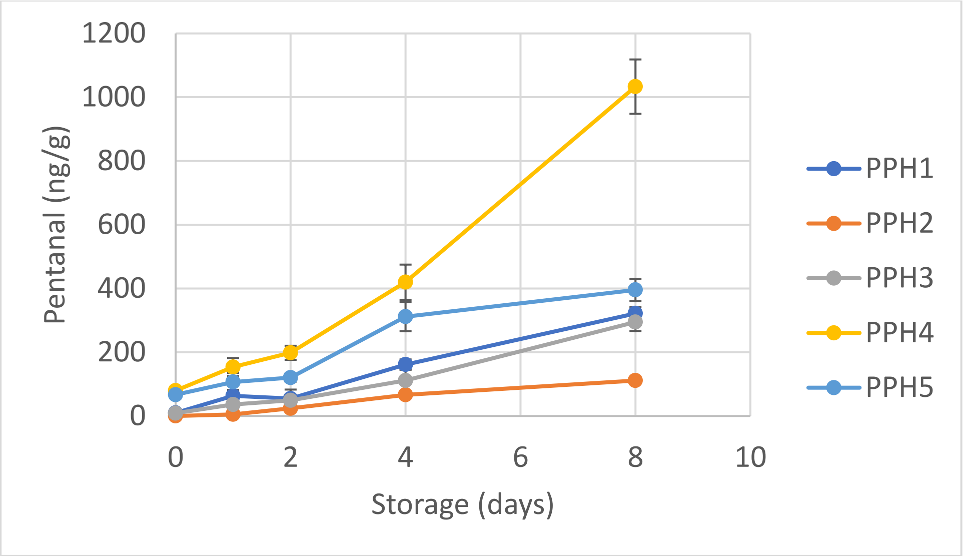

Pentanal (ng/g)
1200
1000
800
600
400
200
0
0
2
4
6
8
10
Storage (days)
PPH1
PPH2
PPH3
PPH4
PPH5

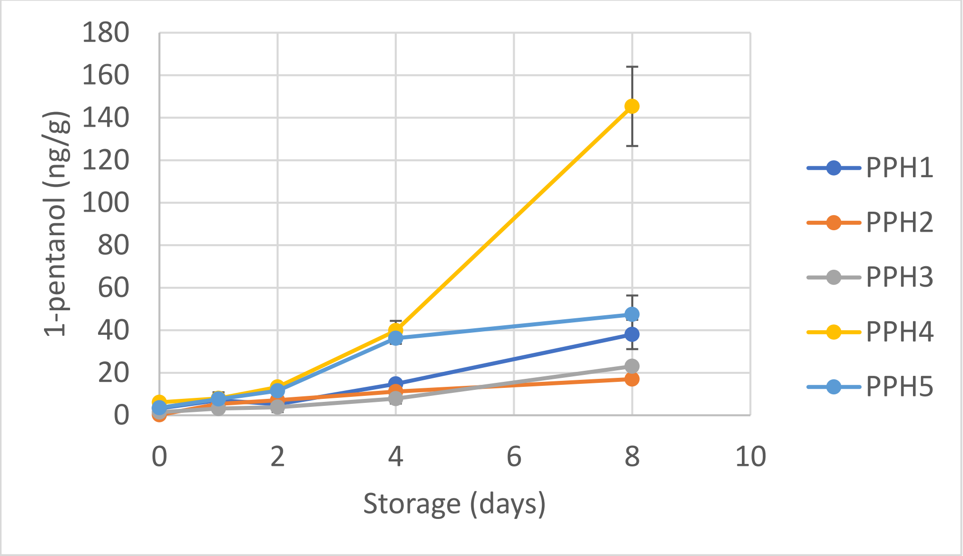

1-pentanol (ng/g)
180
160
140
120
100
80
60
40
20
0
0
2
4
6
8
10
Storage (days)
PPH1
PPH2
PPH3
PPH4
PPH5

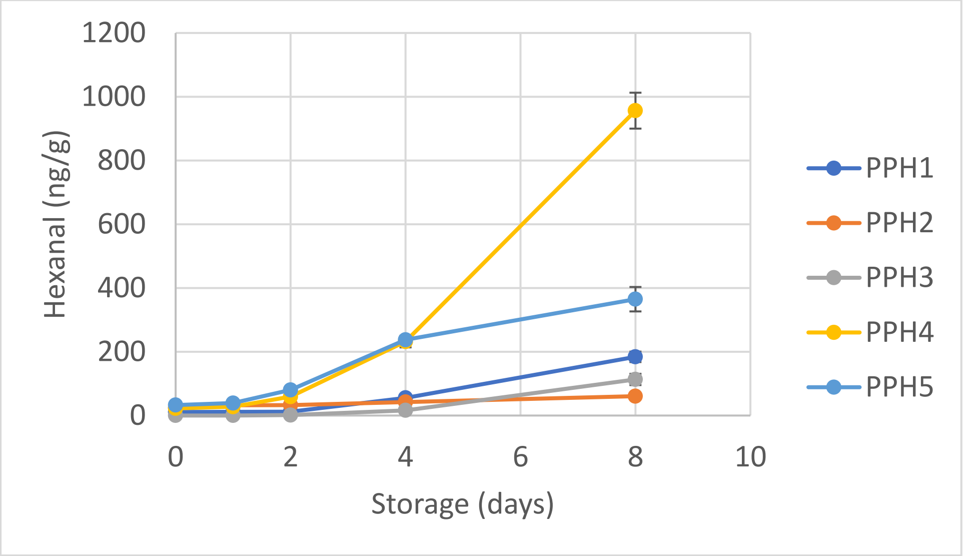

Hexanal (ng/g)
1200
1000
800
600
400
200
0
0
2
4
6
8
10
Storage (days)
PPH1
PPH2
PPH3
PPH4
PPH5

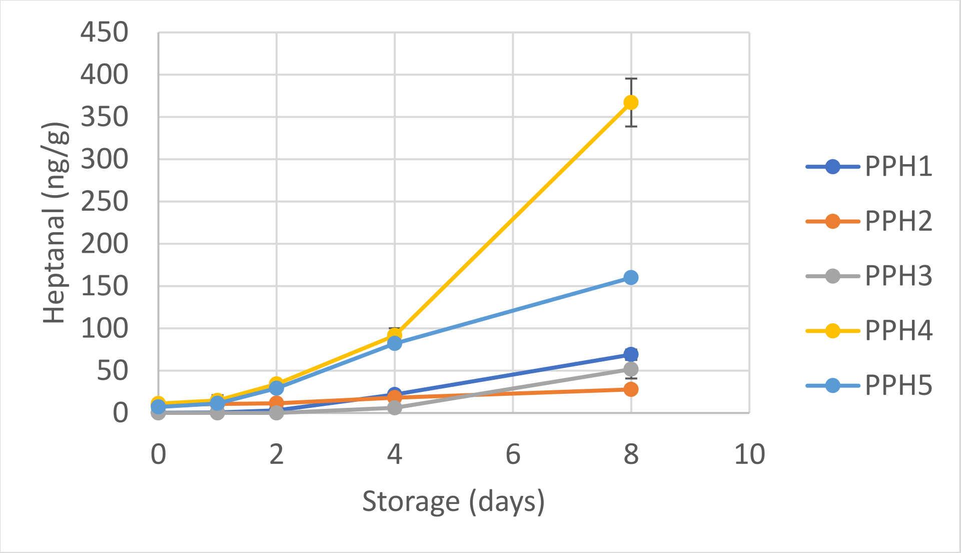

Heptanal (ng/g)
450
400
350
300
250
200
150
100
50
0
0
2
4
6
8
10
Storage (days)
PPH1
PPH2
PPH3
PPH4
PPH5

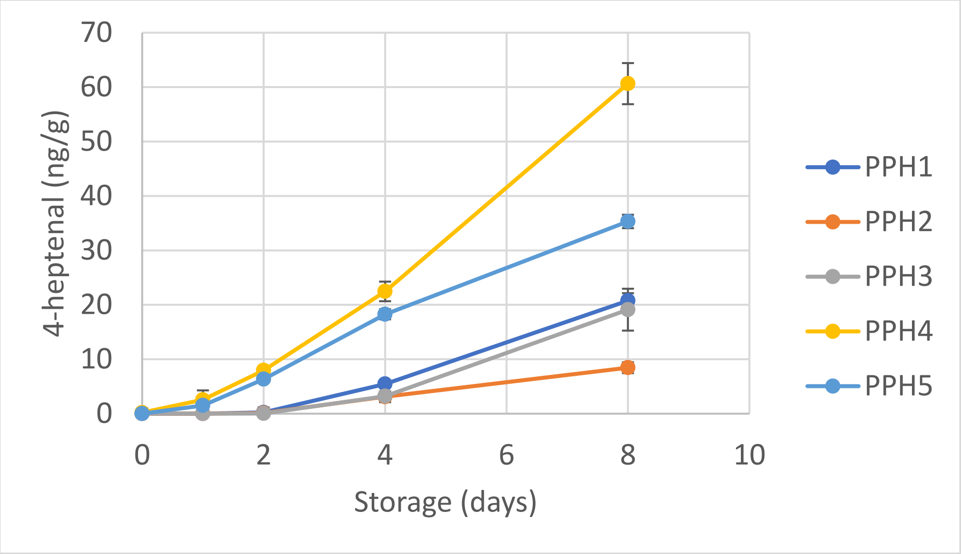

4-heptenal (ng/g)
70
60
50
40
30
20
10
0
0
2
4
6
8
10
Storage (days)
PPH1
PPH2
PPH3
PPH4
PPH5

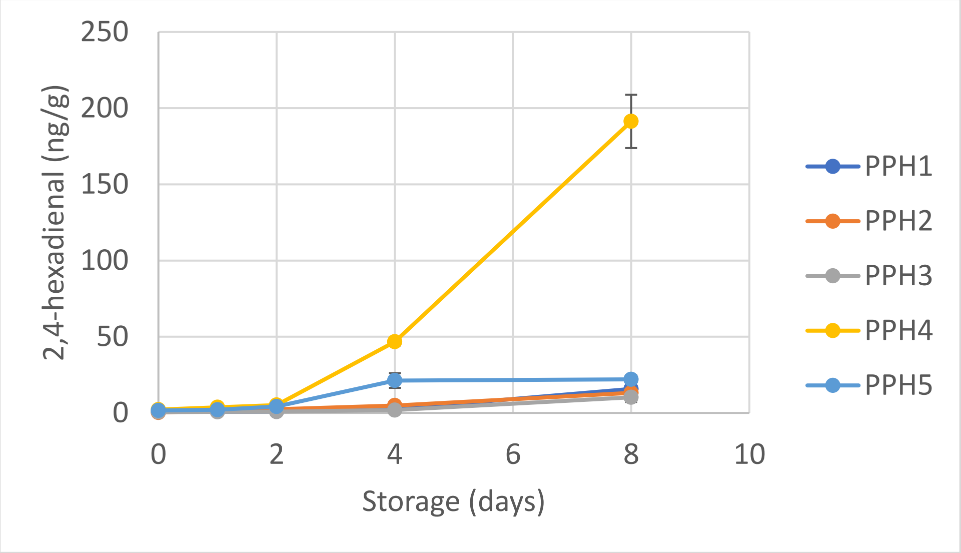

2,4-hexadienal (ng/g)
250
200
150
100
50
0
0
2
4
6
8
10
Storage (days)
PPH1
PPH2
PPH3
PPH4
PPH5

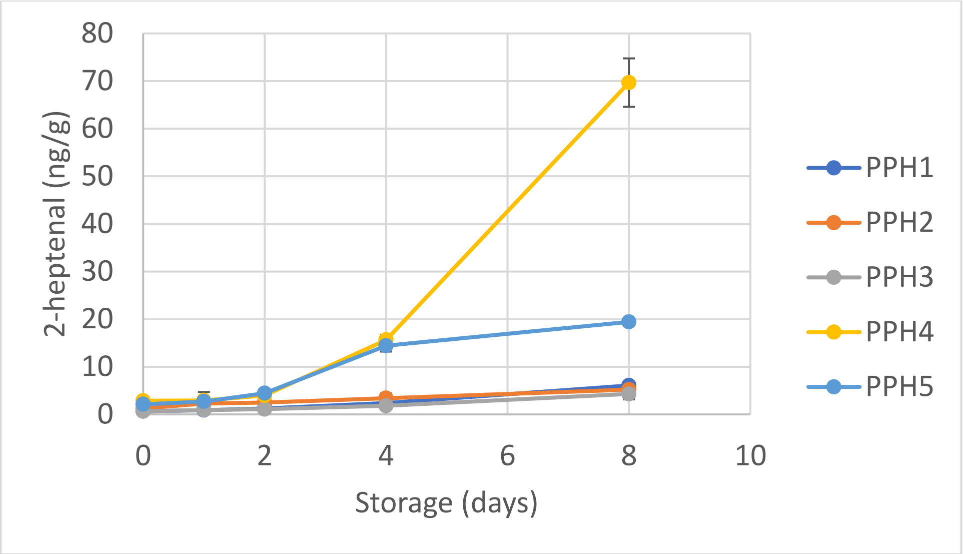

2-heptenal (ng/g)
Storage (days)
PPH1
PPH2
PPH3
PPH4
PPH5

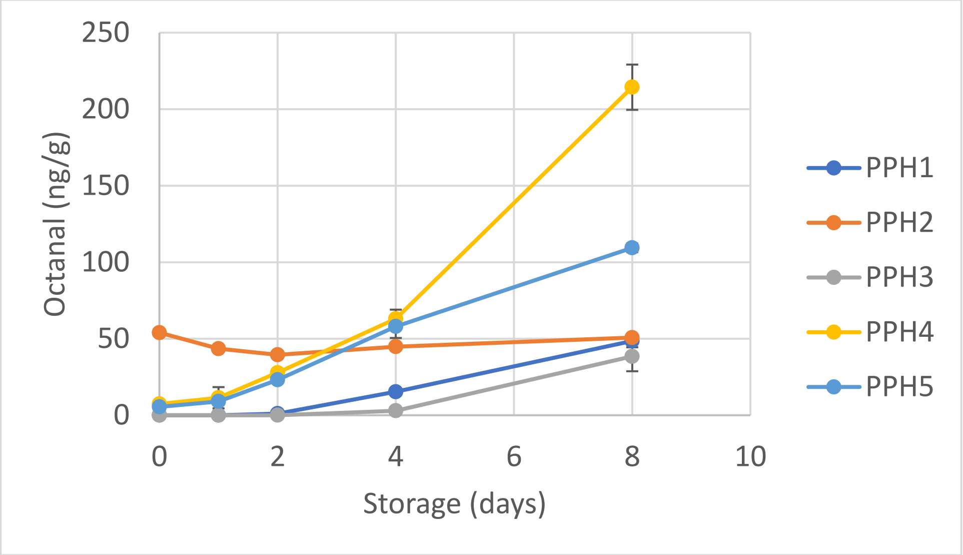

Octanal (ng/g)
Storage (days)
PPH1
PPH2
PPH3
PPH4
PPH5

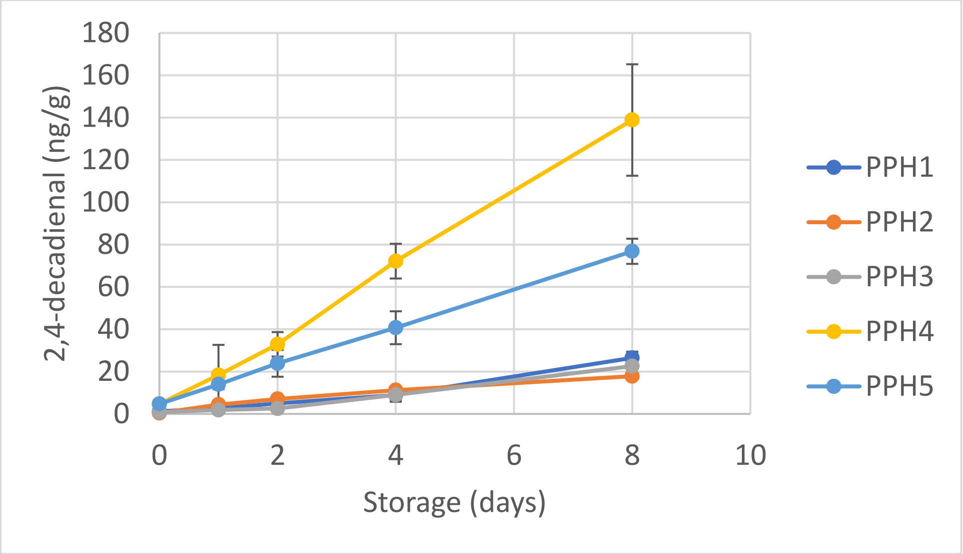

2,4-decadienal (ng/g)
Storage (days)
PPH1
PPH2
PPH3
PPH4
PPH5

Table S3. References for sensory analysis.

ISO 13300-2: 2006 Sensory analysis – General guidance for the staff of a sensory evaluation laboratory – Part 2: Recruitment and training of panel leaders
ISO 3972:2011 & ISO 3972:2011/Cor 1:2012 Sensory analysis – Methodology – Method of investigating sensitivity of taste
ISO 5492:2008 Sensory analysis – Vocabulary
ISO 6658:2017 Sensory analysis – Methodology – General guidance
ISO 8586:2012 Sensory analysis – General guidelines for the selection, training and monitoring of selected assessors and expert sensory assessors
ISO 8589:2007 Sensory analysis – General guidance for the design of test rooms (EN ISO 8589:2010)
NMKL Procedure No. 6 General guidelines for quality assuring sensory laboratories (2023)
